# First drug-checking study at an electronic festival and fentanyl detection in the central region of Mexico

**DOI:** 10.1186/s12954-023-00905-8

**Published:** 2023-12-06

**Authors:** Silvia L. Cruz, Miguel Bencomo-Cruz, María E. Medina-Mora, Fabiola Vázquez-Quiroz, Clara Fleiz-Bautista

**Affiliations:** 1 Department of Pharmacobiology, Center for Research and Advanced Studies (Cinvestav), Mexico City, Mexico; 2 Opioids Working Group. Global Studies Seminar, Faculty of Medicine, National Autonomous University of Mexico (UNAM), Mexico City, Mexico; 3 Substance Analysis Program-Deliberar A. C. and ReverdeSer Collective A. C., Mexico City, Mexico; 4 Faculty of Psychology Director, National Autonomous University of Mexico, Mexico City, Mexico; 5 National Institute of Psychiatry Ramon de la Fuente Muñiz (INPRFM), Calzada México-Xochimilco 101 Col. San Lorenzo Huipulco, 14370 Mexico City, Mexico

**Keywords:** Festival, Young people, Adulterants, Fentanyl, Stimulants, Drug-checking

## Abstract

**Background:**

Perception of drug adulteration has increased in Mexico, but there is little research on adulterants and toxicity. The aim of this study was to identify drug composition in an electronic music outdoor festival nearby Mexico City.

**Methods:**

The participants completed a questionnaire with demographic data, harm reduction strategies, drug-use patterns, history, and the drug they expected to find. We took a small sample of each substance and prepared it for drug checking. A two-section drug testing station was placed within the grounds of the festival. Interaction with participants occurred at the front part. Drug checking was conducted at the rear part. The service was free of charge, voluntary and confidential. Forty persons aged 22 to 48 years participated (mode = 28), of which 92.5% were male, most (82.5%) were single. Through the Substance Analysis Program of “ReverdeSer Collective,” we conducted the testing with the attendants that provided 51 drug samples, following ethical and biosafety protocols. We used colorimetry, Fourier Transform Infrared Spectroscopy, and fentanyl immunoassay strips for sample analysis.

**Results:**

Substances of choice among attendants were psychostimulants (MDMA and other amphetamine-like drugs) and hallucinogens. Most samples contained what the users expected plus adulterants. Main adulterants were methylene-dioxy-ethyl-amphetamine, methylene-dioxy-propyl-amphetamine, hydroxyamphetamine, and the selective serotonin reuptake inhibitor venlafaxine. Fentanyl was present in 2 out of 4 cocaine samples and in 14 of the 22 confirmed MDMA samples.

**Conclusions:**

Some of the adulterants found pose serious health risks, especially fentanyl, amphetamine-like substances, and venlafaxine. Therefore, it is urgent to monitor these adulterants at electronic music festivals and to implement prevention, treatment, and harm reduction public policies. Naloxone distribution and drug-assisted therapies should be part of government programs in Mexico.

## Article Highlights

- We used FTIR for drug checking at a music festival close to Mexico City
- We also analyzed drug samples with fentanyl test strips and UV light
- 51 samples, mostly nominal MDMA and LSD, from 40 users were analyzed
- Most had the expected active substance plus adulterants, including venlafaxine
- We detected fentanyl in MDMA and cocaine samples.

## Background

The current international drug policy criminalizes people who use drugs (PWUD) and illicit markets offer adulterated substances that can cause serious health consequences [1]. Drug sellers augment volume and revenue by adding inert and active adulterants to drug samples. Pharmacologically active adulterants are usually synthetic drugs or over-the-counter medicines. Some examples are caffeine, local anesthetics (e.g., benzocaine, lidocaine), non-steroidal anti-inflammatory analgesics (e.g., phenacetin, acetaminophen), veterinary drugs (e.g., levamisole), fentanyl and its analogs [2, 3]. Other adulterants are new psychoactive substances (NPS) not controlled by the International Narcotics Control Board, such as NBOMe, an LSD substituent [4]. Fatal overdose risk increases when potent opioids are mixed with heroin, cocaine, and methamphetamine (crystal Meth).

Electronic music outdoor festivals (EMOFs) congregate young people vulnerable to risk-taking behaviors, including drug use. Drug-checking services (DCS) are present at festivals in Canada [5], Spain [6], Netherlands [7], Portugal [8], Austria [9], Switzerland [10], United Kingdom [11], Australia [12], and Colombia [13]. These services reduce drug use harms by giving information to PWUD about the risks associated with using “typical” doses of known drugs and alert on the presence of adulterants [14].

DCS are scarce in Mexico. The Substance Analysis Program (SAP), supported by the Institute for Attention Care and Prevention, the National Human Rights Commission, and the NGO “ReverdeSer Collective” initiated operations in 2014 [15]. Between 2015 and 2019, SAP collected 1585 samples from 1407 drug users in 28 EMOFs in Mexico City and surrounding cities. Using colorimetric analysis, thin-layer chromatography, and UV light analysis, SAP found that LSD and MDMA accounted for 90% of the drugs tested. MDMA was frequently mixed with crystal Meth, and LSD was adulterated with, or substituted by, NBOMe. Cocaine constituted only 2% of the drugs tested, but most samples (80%) had several adulterants [16].

Sensitive fentanyl test strips have been used to detect fentanyl and fentanyl derivatives in the paraphernalia of heroin and crystal users in Tijuana, a northern border city of Mexico. The results showed that 93% (55 out of 59) of white powder samples sold as heroin (known as *China white*) were laced with fentanyl [17]. Another recent study detected fentanyl in almost 53% (*n* = 652) of the paraphernalia collected at various harm-reduction places in Tijuana [18].

Considering the increasing occurrence of adulterants, and the dangers associated with them, the aims of this study were (a) to identify the drug composition of substances voluntarily provided by attendants to an EMOF in a place nearby Mexico City, using FTIR; (b) to compare the substances found in samples with those expected by drug users; (c) to determine if fentanyl was present in the collected samples; and (d) to characterize the consumption history and consumption effects of the participants.

## Methods

### Data collection

Samples were collected at the EMOF from 15:00 to 24:00 h as part of the SAP’s harm reduction program. The DC service was free of charge, voluntary, and confidential. It was conducted at a mobile drug testing station within the grounds where the festival took place. We provided consent forms to participants, asked them to sign with a moniker to keep anonymity, and gave information of the procedure to analyze their samples. The attendants were asked to complete a questionnaire, including demographic data, history, patterns of drug use, and harm reduction strategies. They also mentioned which drug they expected to find. A team member collected a small sample of each substance and annotated its physical characteristics (shape, color, and weight), along with the intended administration route. While users answered the questionnaire, another team member divided each sample into four parts (approximately 5 mg each), three for colorimetric analysis and one for the FTIR spectrometer. We wore latex gloves to avoid skin contact and handled drugs with stainless steel tweezers and spatulas. Tools were cleaned between samplings to prevent contamination. Drug identification was conducted in a separate but contiguous section of the drug-checking station.

### Drug testing

To identify LSD, we cut blotters’ outer edges or a small gummy sample, placed them into individual 1.5 ml Eppendorf tubes with 1 ml methanol, stirred the vials, and examined the samples under UV light. Blue fluorescence confirmed LSD presence [15]. Powders, granules, and pills were analyzed with colorimetry, the FTIR spectrometer, and fentanyl immunoassay strips. For colorimetric analyses, we used Markis, Mecke, and Mandelin reagents and a reference color card for drug identification [19, 20]. Colorimetric testing allows identification of common drug groups rather than individual substances. Examples of groups are MDMA/MDA/MDE, amphetamine/methamphetamine, or NPS hallucinogens (DOB, DOM). We also used a desktop Fourier Transform Infrared Spectrometer (FTIR, Bruker Alpha II) with a platinum-attenuated total reflectance (ATR) module and a diamond ATR crystal, OPUS software and the TICTAC ATR-FTIR and ATR-PHARMA libraries, which contain the spectra of common drugs and adulterants. The FTIR spectrometer detects the components of mixed samples, providing rapid qualitative and quantitative results with a lower detection limit of approximately 5% [21].

After FTIR analysis, we separated a tenth or less of the sample with a spatula, added 0.5 ml of water using a transfer pipette, recovered the liquid, and applied a small amount to the fentanyl testing cassette (Certum Diagnostics; detection limit: 20 ng/ml, with specificity equivalent to BTNX strips; [17]. One red line meant a positive results; two lines, a negative result. Each sample analysis took approximately 10 min. A team member with knowledge of substances’ effects and harm reduction strategies, and experience in counseling work delivered the results to each participant and explained the possible risks of experiencing unwanted effects. The Ethics Committee of the Ramon de la Fuente Muñiz National Institute of Psychiatry approved this protocol (Protocol Number CEI C/057/2021).

### Data analysis

We used version 21 of the SPSS program for descriptive data analysis and GraphPad Prisma 8.2.1 to calculate Fisher’s exact test.

## Results

### Users and expected substances

We collected 51 samples from 40 users, all of whom completed the required questionnaire. Data analysis provided the following information:

#### Sociodemographic characteristics

The age range was from 22 to 48 years (mean = 30, mode = 28), 92.5% were male and most (82.5%) were single.

#### Reasons for requesting the drug-checking service

In response to the question concerning the reasons to check their substances, most users mentioned their health, and more than half explicitly said that they wanted to know the actual sample composition. In addition, approximately a third of them considered drug-checking an opportunity to learn about drug effects (Table 1).

**Table 1:** Reasons for requesting drug checking

Reasons	%
I care about my health	75.0
To know the actual drug composition	67.5
I care about the quality of the substance	65.0
I want to know if my substance is adulterated	60.0
I care about my friends' health	42.5
Curiosity	40.0
To know the substance's effects	32.5

#### Expected substances, providers, and costs

Drug samples were expected to be psychostimulants and hallucinogens: MDMA (64.7%), LSD (17.6%), cocaine (9.8%), ketamine (3.9%), methamphetamine (2%) and DMT (2%). Users obtained their substances through dealers (73.5%), friends (14%), or as gifts (10%). Most acquired them at Mexico City (70%) or neighboring cities, including the State of Mexico (8.2%), Morelos (6.1%), Queretaro (6%), and Aguascalientes (2%). The substances were crystals (39.2%), powders (29.4%), blotters (15.7%), pills (11.8%), a gummy jelly (2%) and one capsule (2%). The prices varied from approximately 5 to 80 USD, with the highest cost corresponding to MDMA.

#### Drug use history

Most customers had a history of substance use, with more than 80% being frequent cannabis users. Above 60% had used MDMA and LSD. In the month preceding the festival, more than two-thirds used cannabis, approximately a third used cocaine and LSD, and 45% used MDMA (Table 2). Substances less frequently used included methamphetamine and 2-CB. Only 4% and below used ketamine or mushrooms in the preceding month.

**Table 2:** Percentage of users reporting past-year and past-month drug use

Drug used	Past year	Past month
Cannabis	85	75
MDMA	72.5	45
LSD	60	30
Cocaine	42.5	27.5
Methamphetamine	17.5	7.5
2-CB	10	10

As to the use of substances subjected to checking, more than half of the respondents (56.6%) said it was occasional (1 to 2 times in the last 3 months), 39.1% said it was frequent (1 to 3 times per month in the last 3 months), and 4.3% used it very frequently (also known as weekly: 1 to 4 times per week in the last 3 months). Most participants associated EMOFs with MDMA and hallucinogen use. Almost a third (36.4%) said they provided half of their dose for checking because they had already used the other half. In response to the possibility that their substance would turn out to be different from what they expected, 21.9% said that they would use it regardless of the results. Most participants reported having ever experienced unwanted side effects (Table 3). The most common adverse experience was anxiety (in up to 70% respondents), followed by “nervousness” (possibly another form to call anxiety), insomnia, sweating, distress, and paranoia. Tachycardia and loss of appetite occurred in approximately half of the participants, which is consistent with the use of psychostimulant substances. Some unwanted effects, like forgetfulness, disorientation, depression or anxiety could be sequels rather than acute effects, but this was difficult to discern with the survey used. Future studies should include a specific question about residual unwanted effects. An important result of this study is that five participants (12.5%) had overdosed, two with MDMA, one with DMT, another with NBOMe, and the last one with an unidentified substance.

**Table 3:** Percentage of users with history of unwanted side effects

Effects	%
Anxiety	70.0
Insomnia /Sweating	65.0
Nervousness	60.0
Distress, Paranoia	55.0
Tachycardia / Nausea, Vomiting	50.0
Loss of appetite	45.0
Disorientation	40.0
Oversights, forgetfulness /Depression	35.0
Tremors	30.0
Involuntary movements /Hallucinations /Lethargy	25.0
Overdosing	12.5
Sore throat	10.0
Fever / Shortness of breath	5.0

#### Harm reduction information

Approximately two-thirds of the subjects (62.5%) were familiar with harm-reduction programs. All participants said that these services should be public, widely available and steadily promoted; 37.5% had previously used a drug-checking service.

### Drug checking results

#### Most samples contained the expected active substance

**Fig. 1:**
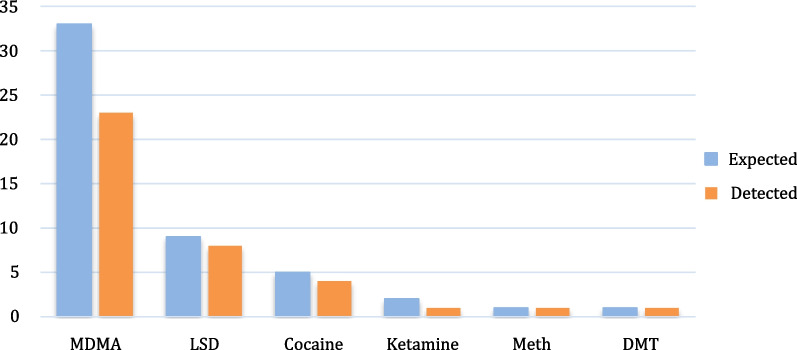
Number of samples expected and confirmed to be MDMA

FTIR analysis confirmed that most samples contained what the user expected (Fig. 1), and UV analysis confirmed that 8 out of 9 “LSD” samples were positive for that substance. Colorimetric testing accurately identified MDMA and amphetamine-like drugs, but not MDMA analogs.

#### Most substances were laced with other drugs

**Fig. 2:**
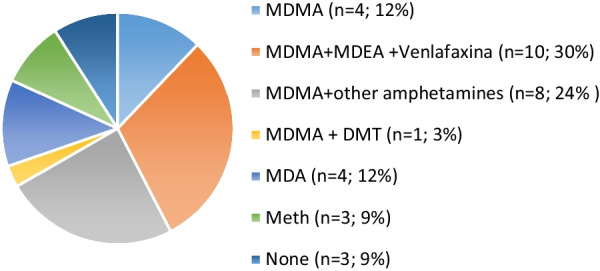
Percentage and number (in parenthesis) of substances detected in “MDMA” samples

Only 4 of the 33 nominal MDMA samples had MDMA without active adulterants (contents ranging from 31.2 to 100%), 10 did not have MDMA, and 19 had MDMA combined with other psychoactive substances, including the closely related amphetamines methylene-dioxy-ethyl-amphetamine (MDEA), methylene-dioxy-propyl-amphetamine (MDPA), hydroxyamphetamine, the hallucinogen dimethyltryptamine (DMT), and the selective serotonin reuptake inhibitor (SSRI) venlafaxine (Vlx). Interestingly, the specific MDMA + MDEA + Vlx combination accounted for almost a third of nominal MDMA samples. Among those lacking MDMA, 4 had MDA (38.5–63.7%), 3 had crystal Meth (14–62.3%), and 3 did not have any psychoactive substance (Fig. 2). Of these, one had lactose, another had rubber carbon, and the third had a cellulose derivative (hydroxybutyl methyl cellulose) and sodium salts.

In general, the content and type of adulterants varied greatly among samples, both in type and content (Table 4). In a few cases, they completely substituted for the expected substances (e.g., benzocaine as a cocaine substituent and MDA or crystal Meth instead of MDMA).

**Table 4:** Adulterants found in the samples and their content (by percentage)

Adulterants/Substituents	*n*	[Range]	(Mean ± s.d.)
MDEA	15	[15–50.8]	28.7 ± 10.3
Venlafaxine	10	[13.1–24.1]	19.1 ± 3
MDA	5	[38.5–63.7]	49.2 ± 10.3
Meth	3	[14.2–100]	58.8 ± 43
MDPA	3	[9.4–29]	18 ± 8.2
Desoxyephedrine	2	[9.9–23.4]	16.7 ± 9.5
DMT	2	[17–61.4]	39.3 ± 31.5
Lactose	2	[40.9–58.5]	49.7 ± 12.4
Benzocaine	1	100	
Caffeine	1	43.0	
Hydroxyamphetamine	1	9.0	
Methyl cellulose	1	26	
Pentoxifylline	1	21.0	
Rubber carbon	1	66.9	
Sodium salts	1	46.6	

#### A significant proportion of the samples tested positive for fentanyl

None of the users expected to find fentanyl in their substances, but 14 out of the 22 confirmed MDMA samples and 2 out of 4 cocaine samples tested positive for this substance. Knowing that false positives can occur in concentrated MDMA samples, we analyzed if there was a correlation between fentanyl positivity and high NMDA concentration, but the result (*p* = 0.167; Fisher’s exact test) did not reach statistical significance (Table 5).

**Table 5:** Positive fentanyl cases in samples with different MDMA concentrations

Fentanyl	NMDA concentration		Totals
	< 30%	> 30%	
Positive	7	7	14
Negative	7	1	8
Totals	14	8	22

## Discussion

The results of this study show that the substances of choice among attendants were psychostimulants and hallucinogens. Most of the samples contained what the users expected. MDMA was frequently combined with, or substituted by, closely related amphetamines, such as MDA, MDPA, or MDEA. Nominal LSD was indeed LSD in eight out of nine samples. Attendants to the festival were reassured by confirming that their substance was what they were offered and bought but surprised by the presence of fentanyl and venlafaxine and uncertain of the risks associated with their consumption.

### Characteristics of DCS users

All users recommended the service, would use it again, and considered that they can prevent bad experiences and fatal outcomes. Festivals attendants in Australia [12] and Europe [22] have expressed similar opinions, showing a service acceptancy of 94% and 87%, respectively.

Almost all who approached the DCS were men (92.5%). Possibly women used less substances than men, preferred their partners to ask for the testing, or did not want to be identified as users. Actions with a gender perspective should be implemented to make them feel confident to test their substances in an informed, free, and autonomous manner. This could reduce the adverse effects and risky situations, including sexual or other types of physical violence.

Most DCS attendants used more than one substance per occasion. In our study, 11 out of 40 users provided two different samples for analysis. Studies conducted in other EMOFs have also reported polysubstance use, either by combining substances with similar profiles (e.g., psychostimulants) or from different groups, such as psychostimulants and hallucinogens [23, 24]. A report from Zurich found that 19% of EMOF attendants combined MDMA with cocaine, and 22% preferred to use MDMA with other amphetamines. However, the same report mentioned that 81% of the population regularly used psychostimulants, alcohol, and cannabis [21]. Another recent study showed that 12% of festival attendants in the USA are polydrug users who mix cannabis, cocaine, and amphetamine-like substances [25]. In our sample, most DCS users reported previous and last-month use of cannabis and MDMA.

### On-site DCS experience

Previous studies have already mentioned the advantages of using complementary techniques for drug checking [5]. Simple colorimetric methods allow for rapid drug screening of drug groups [26]; however, they cannot identify adulterants [22]. FTIR can, which is why it has been included in several DCS [27, 28]. However, FTIR cannot detect potent substances like fentanyl, because they are present in very low concentrations. Because of this, we used fentanyl immunoassay strips. These strips were designed to identify fentanyl and its analogs in urine, but have been used for drug checking in paraphernalia [17] and drug samples at EMOFs [5], applying some caution measures because false negatives can occur if fentanyl is unevenly distributed in a sample, and false positives when samples have high concentrations of MDMA or other amphetamine-like drugs [29]. Using fentanyl strips and gas chromatography coupled with mass spectrometry (GC/MS), Lockwood and coworkers found false positives in samples containing MDMA, crystal Meth, or diphenhydramine at concentrations equal to or above 1 mg/ml [30]. Similarly, a recent study found 9.6% false negatives and 3.7% false positives with fentanyl strips in previously analyzed samples [31]. Although all methods have limitations, combining them provides better results [21].

### Adulterants

Common adulterants can be contaminants used in the process of synthesis or handling of the drug (e.g., solvents, precursors); biological contaminants; inert diluents that increase the volume of the samples; or pharmacologically active adulterants, which are added to the drugs to alter their psychoactive effects. Except for biological contaminants, we found all types of substances in the samples analyzed. Desoxyephedrine is a precursor used to synthesize amphetamine-like drugs; lactose, methylcellulose, rubber carbon, and sodium salts are inert diluents, and many MDMA adulterants are amphetamine-like drugs.

Several studies in Europe and the USA that have found mephedrone, methylone, and new psychoactive amphetamine derivatives, as main MDMA adulterants [20, 32]. MDA has also been detected in oral fluids from MDMA users, along with ethylone and methylone [33]. Our MDMA samples were mixed with amphetamine-like substances, mainly MDA, MDAE, and crystal Meth, but not with NPS, which is consistent with the only other report available from Mexico [16]. Venlafaxine was present in a third of our confirmed MDMA samples. To our knowledge, there are no reports on the presence of this substance as an MDMA adulterant. Venlafaxine is a selective serotonin reuptake inhibitor (SSRI) used as an antidepressant and anti-anxiety medication, and, as such, it increases serotonin circulating levels. MDMA and MDMA-like drugs also increase serotonin release [34]. Because of this, combining venlafaxine with MDMA-like drugs could enhance both the psychoactive and adverse side effects. In this regard, a recent study found that the odds of fatal outcomes were significantly higher in people who were under venlafaxine medication and used MDMA [35]. Although life-threatening cases are rare, this particular combination can increase the risk of serotonin syndrome occurrence [36], which is a medical emergency characterized by tremor, hyperreflexia, diaphoresis, hypertonia and hyperthermia [34].

On the other hand, results obtained from several DCS in Europe from 2008 to 2013 found levamisole is the main cocaine adulterant, followed by phenacetin and local anesthetics [32]. We only found benzocaine, a local anesthetic, as cocaine substituent, but the number of confirmed cocaine samples was small (4 out of 5). Of interest, two of them tested positive for fentanyl together with a high proportion of our MDMA samples. This contrasts with the results found at an electronic music festival in Canada using fentanyl strips, where only 31 out of approximately 1000 samples were positive [37]. As previously mentioned, high concentrations of MDMA and amphetamine-like substances can yield false positives [29–31], and we could not conduct a GC/MS analysis to confirm our findings. Despite the likelihood of fentanyl overestimation, it is worth noting that we did not find a significant correlation between positive tests and high MDMA concentration. Moreover, a 100% MDMA sample was negative for fentanyl, and a 12.9% MDMA was positive. These data, together with the two cocaine samples that tested positive for fentanyl, provide evidence of fentanyl presence in a place close to Mexico City. Present results show that fentanyl adulteration is no longer a phenomenon confined to the northern border of Mexico among vulnerable people who inject heroin or crystal Meth, but has reached young people who use psychostimulants.

The intentional use of cocaine combined with opioids (usually heroin) is a practice known as “speedballing” [38, 39], while “goofballing” refers to using methamphetamine with heroin or fentanyl [40]. Opioids and stimulants have different mechanisms of action. Cocaine blocks dopamine and norepinephrine reuptake, thus increasing excitatory neurotransmitter levels. Amphetamines promote the indirect release of dopamine, norepinephrine, and serotonin, also having a stimulant effect. Opioids have a complex mechanism of action [41], which includes blocking calcium entry and increasing potassium output, making cells less responsive to neuronal stimulation. Despite these differences, psychostimulants and opioids share the ability to produce rewarding effects by increasing dopamine levels in the mesolimbic brain system and producing similar neuroadaptation in the brain reward system [38, 41].

Motives to combine opioids and psychostimulants include increasing the opioid psychoactive effect, decreasing the excessive stimulation caused by cocaine or amphetamines, and diminishing the intensity of opioid withdrawal [42]. People who sell substances have replaced heroin with fentanyl due to its high addiction liability and large profit margin.

In addition, the observational register during data collection yielded some hypotheses that could explain the findings on the adulteration of cocaine with fentanyl: 1) a significant proportion of the young people who attend these types of festivals belong to middle and high socioeconomic strata, which allows them to have access to higher-cost substances such as cocaine, whose price can reach 60 dollars per dose; 2) this population has a recreational and occasional use, seeking to prolong the effects of the substances, hence their preference for the ones reported in this study. For the groups dedicated to the illicit production of fentanyl, reaching other populations, supplying it in more regions of Mexico and adulterating drugs different to heroin, can be highly profitable; 3) the purchase and distribution of the substances in many cases is done through social networks, neither the final dealers nor the population are aware of the origin of the drug acquired, much less of the cutting agents present in them. Likewise, we observed that, although the young people who attended the festival had a high degree of education and some information about the effects of certain drugs, they did not have sufficient knowledge of fentanyl, and neither did they intentionally seek it out, and despite their capacity for agency to manage some risks associated with the use mainly of stimulants, their high normalization and low perception of risk regarding drug use, places them in a vulnerable situation particularly in the face of fentanyl. These data indicate that it is very important to implement prevention and risk reduction programs, as well as to design communication strategies about this opioid addressing young population [43].

### Study limitations

The sample size was small, nonrandom, and collected during a single EMOF; therefore, data cannot be generalized to other populations. We used a minimal amount of each drug diluted in a relatively small volume of water; it would be better to use higher volumes in future studies to prevent the possibility of false fentanyl-positive tests in MDMA samples. Finally, we could not verify our results with GS/MS.

## Conclusions

Substance analysis is a strategy to monitor new drug markets in different street contexts, such as festivals and meeting places where people may be exposed to adulterated substances. Harm reduction programs and interventions in such environments can inform users about the content of their substances and the possible adverse effects of adulterants. Despite its limitations, the present study provides a snapshot of the type of substances available at a trendy Mexican festival. Some of the adulterants found in our study have serious health risks. This finding is an early warning to monitoring their possible extension to other population groups and regions outside the northern border of Mexico [17]. Early alert systems at festivals should exist to alert on the presence of fentanyl or other dangerous combinations (such as the MDMA/MDEA/venlafaxine here reported), as it occurs in other countries [44]. Fentanyl overdoses are life-threatening and serotonin toxicity is a medical emergency that requires specialized on-site treatment. Control of all variables in outdoor festivals can be challenging, but having access to the opioid antidote naloxone, and medical assistance to assist intoxicated people can prevent life losses.

## Data Availability

The datasets used and/or analyzed during the current study are available from the corresponding author on reasonable request.
